# Development of Principles for Health-Related Information on Social Media: Delphi Study

**DOI:** 10.2196/37337

**Published:** 2022-09-08

**Authors:** Emily Denniss, Rebecca Lindberg, Sarah A McNaughton

**Affiliations:** 1 Institute for Physical Activity and Nutrition School of Exercise and Nutrition Sciences Deakin University Melbourne Australia

**Keywords:** social media, health information, information quality, quality assessment, research tool, credibility indicator, credibility, credible, eHealth, Delphi, social media, assessment tool, measurement tool, web-based information, mobile phone

## Abstract

**Background:**

Health-related misinformation can be propagated via social media and is a threat to public health. Several quality assessment tools and principles to evaluate health-related information in the public domain exist; however, these were not designed specifically for social media.

**Objective:**

This study aims to develop Principles for Health-related Information on Social Media (PRHISM), which can be used to evaluate the quality of health-related social media content.

**Methods:**

A modified Delphi approach was used to obtain expert consensus on the principles and functions of PRHISM. Health and social media experts were recruited via Twitter, email, and snowballing. A total of 3 surveys were administered between February 2021 and May 2021. The first survey was informed by a literature review and included open-ended questions and items from existing quality assessment tools. Subsequent surveys were informed by the results of the proceeding survey. Consensus was deemed if ≥80% agreement was reached, and items with consensus were considered relevant to include in PRHISM. After the third survey, principles were finalized, and an instruction manual and scoring tool for PRHISM were developed and circulated to expert participants for final feedback.

**Results:**

A total of 34 experts consented to participate, of whom 18 (53%) responded to all 3 Delphi surveys. In total, 13 principles were considered relevant and were included in PRHISM. When the instructions and PRHISM scoring tool were circulated, no objections to the wording of the final principles were received.

**Conclusions:**

A total of 13 quality principles were included in the PRHISM tool, along with a scoring system and implementation tool. The principles promote accessibility, transparency, provision of authoritative and evidence-based information and support for consumers’ relationships with health care providers. PRHISM can be used to evaluate the quality of health-related information provided on social media. These principles may also be useful to content creators for developing high-quality health-related social media content and assist consumers in discerning high- and low-quality information.

## Introduction

### Background

Health information–seeking behavior can influence an individual’s decision-making and overall health [1]. The internet is a popular source of health-related information for the general public [2-5], and its popularity has been growing [6]. The internet has facilitated web-based environments where information can be published and accessed with tremendous speed and ease [7]. Technological innovations have led to the widespread use of devices such as smartphones, tablets, and computers, especially in high- and middle-income countries, and consumer access to the internet and information is now ubiquitous [3,8,9]. Consequently, web-based environments have become highly accessible and efficient sources of health-related information.

Social media is a prominent component of web-based environments. Social media refers to internet-based applications that facilitate user-generated content, allow individuals to create user profiles and identities, and develop web-based networks by connecting user profiles and groups [10]. Each social media platform has unique characteristics; however, all platforms share these common features [10]. Users can amass large followings, and information in the form of text, images, and videos can be instantaneously published and viewed by many people [10,11]. Approximately half of the global population comprises active social media users, and rates of social media use are steadily and continuously increasing [12]. As social media has grown in popularity, so too has its use by consumers for health-related information [13,14]. Consumers not only actively seek health-related information on social media but are also passively exposed to it in their social media feeds [15,16].

The spread of health-related misinformation on social media has been identified as a serious threat to public health [17-19]. All social media users have the freedom to publish and share information on almost any topic, regardless of their credentials, and consequently, information on social media is of variable quality and veracity. In addition, previous research has identified low levels of media literacy among consumers [20], which is the ability to access, understand, and critically evaluate information presented in the media [21]. These factors have combined and contributed to the propagation of health-related misinformation on social media, which has the power to undermine credible public health messaging [17,18]. For example, it is believed that the publication and spread of misinformation on social media have amplified and accelerated the transmission of the SARS-CoV-2 virus [18,22], contributing to decreased vaccination rates and the re-emergence of previously eradicated diseases such as the measles [23,24]. Communication of high-quality health information via social media has potential benefits, such as increased accessibility of information, improved communication between health professionals and patients, and social and emotional support for patients [25]. However, social media health information is generally not of high quality, particularly in relation to cancer, diabetes, and dental care [26].

The quality of health-related information refers to the reliability of information compared against a set of defined quality criteria [27]. There is a large body of literature on health information quality, and many tools and principles have been developed to evaluate the quality of health-related information in specific contexts [27]. The DISCERN instrument [28,29], *Journal of the American Medical Association* (JAMA) benchmarks [30], and the Health on the Net (HONcode) principles [31] are most commonly used to evaluate internet-based health-related information [27]. The DISCERN instrument was established to judge the quality of written health-related information [28], and the JAMA benchmarks and HONcode principles were designed to evaluate and guide the development of information provided on websites [30,31]. These tools were developed by experts, have been extensively used throughout the literature, and have been shown to be reliable and valid measures of information quality across a wide range of health-related topics [27,28,30,31].

Existing quality assessment tools such as the DISCERN instrument, JAMA benchmarks, and HONcode principles and other established tools share common criteria to evaluate health information. Criteria to assess the disclosure of advertising policies, sponsorships, and financial conflicts of interest are included in the most frequently used tools for evaluating internet-based health information [27]. Similarly, existing tools for websites assess whether the date of the information’s publication and last update have been disclosed and whether references to the original sources of information have been included [27]. The commonalities between these tools demonstrate the agreement in the literature regarding the fundamental elements of high-quality health information. Therefore, there is now a need to evaluate the quality of health-related social media content. Thus far, in the literature, studies that have evaluated the quality of social media content have used quality assessment tools designed for different contexts [26]. For example, the DISCERN instrument, designed for written information, and HONcode principles, designed for websites, have both been used to evaluate the quality of YouTube and Facebook content [26]. However, the use of these tools to evaluate social media content may not be suitable, given the unique characteristics of social media and that quality principles from existing tools require adaptation to suit social media.

The widely used DISCERN instrument, JAMA benchmarks, and HONcode principles were developed between 1996 and 1998 and have since undergone minor revisions or have not been updated at all [28-32]. To illustrate some of their limitations in the social media setting, consider that content on social media is usually kept brief to increase user engagement and that some platforms place limits on the number of characters, images, or length of videos in posts [33]. The existing quality assessment tools assume that there are no limits to the length or amount of information provided. As such, it is unlikely that health-related social media content can comprehensively address the quality criteria outlined in these existing tools. In addition, disclosure of funding and conflicts of interest are emphasized in prominent quality assessment tools [28-31]. However, the operationalization of the principles of financial disclosure in previous tools does not consider the covert advertising and influencer marketing that exist on social media and, therefore, may not be sensitive enough to be applied to social media.

The DISCERN instrument, JAMA benchmarks, HONcode principles, and similar tools were developed with static information environments in mind, such as patient information pamphlets, websites, and books. In such environments, the public searches for information to consume it, and experts are better able to act as gatekeepers for credible information [32]. Conversely, in dynamic social media environments, users often consume information passively, and there is an emphasis on user-generated content, which blurs the boundaries between information producers and consumers [32]. As a result, it is more challenging to discern authoritative sources, and previous methods of judging a source’s credibility may lack relevance in the context of social media.

### Objective

There is an increasing number and diversity of social media platforms, and the use of social media for health information–seeking is also increasing. The quality of health information can affect public health communication in both positive and negative directions. As such, the need to measure the quality of health-related social media content has become increasingly important in research settings. Thus far, no quality assessment tool has been developed to suit the particular context of social media. Furthermore, there is a scarcity of literature that has outlined standards for high-quality health-related social media content that can be used to inform the development and selection of credible content. The research gap regarding information quality on social media has also been identified in 2 systematic reviews, which have both described the need for a suitable tool to evaluate the quality of social media content [25,26]. Therefore, the aim of this study was to develop Principles for Health-related Information on Social Media (PRHISM), which defines high-quality health-related information and can be used to evaluate the quality of health-related social media content posted by any public account across all social media channels.

## Methods

### Study Design

This study used a modified Delphi technique, which is a group facilitation method that aims to attain consensus among a panel of experts through iterative surveys and controlled feedback [34,35]. This methodology was selected as it is widely used in health research and is appropriate for facilitating decision-making when there is incomplete knowledge, multiple disciplines involved, or a diversity of opinions on the topic of investigation [34,35]. Furthermore, the Delphi method has been shown to be effective for developing new concepts and is a suitable method for establishing definitions for use in research and practice tools [36-38].

The number of surveys, herein referred to as rounds, was determined a priori [35]. A total of 3 rounds are considered optimal in Delphi studies; therefore, 3 rounds were set to gain consensus and develop PRHISM [35]. Qualtrics software (Qualtrics) was used to host all of the surveys in this study, and surveys were administered between February 2021 and May 2021.

### Ethics Approval

This study was approved by the Deakin University Human Research Ethics Committee (HEAG-H 242_2020).

### Participants and Recruitment

Purposive sampling was used to recruit experts in human health or social media with experience using ≥1 social media platform in a professional capacity for health promotion, research dissemination, or representing a health-related organization. This study used multiple recruitment strategies. Advertising via Twitter was conducted through the accounts of each of the authors and their research institutes based in Melbourne, Australia. An invitation to participate was emailed to the communications departments of 19 Australian and 27 global health organizations that were identified as having a social media presence through Twitter. Snowballing was also used with the recruited participants.

All recruitment materials directed interested parties to a web-based survey to determine their eligibility, register their details, and provide informed consent. To be considered eligible, individuals must have had a minimum of 3 years of experience working anywhere in the world as (1) a health professional registered with a professional body, (2) a researcher or an academic in a health-related field, or (3) a communications or social media specialist in a health-related organization. Such experts were selected as they were familiar with health-related social media content and the characteristics of social media platforms because of their professional experience using social media. Furthermore, it is common for academic institutions, professional bodies, and health-related organizations to have policies or position statements regarding ethical and credible communication on social media. The involvement of eligible individuals with such organizations and the professional use of social media indicated that they were likely to be familiar with issues relating to credible and ethical health communication. Recruitment materials and surveys were provided in only English, and participation was voluntary, with no compensation offered.

### Round 1

Round 1 was informed by a review of the literature. Participants were presented with items from 4 tools. The DISCERN instrument, JAMA benchmarks, and HONcode principles were included as they are the most consistently used instruments in research assessing the quality of health-related information on the internet [27]. The Quality Evaluation Scoring Tool (QUEST; developed to assess internet-based health-related information) was also included as it is highly cited, and its more recent development means that it may cover relevant elements that the older tools do not [39]. All 4 tools included items that can be applied to evaluate a source of health information. The source, assessed for accuracy against the items, is ultimately scored to establish its overall quality.

A total of 33 items from the 4 tools were included in round 1. The participants were asked to rate the importance of each item in the context of health-related information provision specifically on social media. Importance was rated on a 5-point Likert scale ranging from *not at all important* to *very important*. Likert scales are highly recommended for rating statements in Delphi studies, and the 5-point scale was chosen as the optimal number of response categories is between 4 and 7 in Delphi research [34,35,40].

Round 1 also included 6 open-ended questions that probed respondents for deeper insights and provided them with the opportunity to provide written comments about the items. At the end of round 1, participants were also asked to suggest principles not already covered in the presented items to include in PRHISM.

### Round 2

Round 2 comprised a second opportunity for rating the original 33 items, with participants having access to the feedback and results from round 1. In addition, participants rated (on the 5-point Likert scale) 9 new principles and 11 comments, both generated from the open-ended questions in round 1. A summary of all verbatim responses to the open-ended questions from round 1 was also provided. Participants were asked to consider the written responses from other group members when responding to the survey. There were no open-ended questions.

Feedback about group responses to Likert scale questions was provided as the median response and IQR of each question. This feedback method for Likert scale questions is consistently recommended in the literature [34,35,40]. Feedback was presented alongside its corresponding question, and as suggested by Trevelyan et al [35], a visual aid, by way of a bar graph, was also provided to assist participants’ understanding of how the group responded to the preceding round. In line with best practice Delphi methods [27], participants were asked to consider how the rest of the group responded before rating the importance of the quality assessment items again.

In addition to the existing 33 items, 9 new principles suggested from round 1 were added for round 2. These principles were proposed by participants in response to an open-ended question in round 1, which asked for suggestions of additional principles to be included in PRHISM. Suggestions of new principles were content analyzed following the procedure outlined by Keeney et al [40]. Responses that were the same or very similar were initially merged by the first author (ED) before all the authors met to discuss. Disagreements regarding the merging of principles were discussed among the authors until an agreement was reached. Participants were asked to rate the importance of the new principles in round 2 on the 5-point Likert scale.

In addition to the quality principles (33 original items and 9 new principles), 11 comments were included, and participants were asked to rate their level of agreement on a 5-point Likert scale ranging from *strongly disagree* to *strongly agree*. These comments were generated from the responses to the remaining open-ended questions from round 1, which were content analyzed, and merged where similar, following the same method that was used for the newly suggested principles. Comments about items from round 1, which could be used to inform adaptations to the principles that may ultimately be included in PRHISM, were provided to participants in round 2.

### Round 3

A total of 22 principles were included in round 3, comprising 13 adapted principles, 6 principles from pre-existing quality assessment tools, and 3 new principles suggested by participants in round 1. Principles (ie, the items) from round 2 achieving consensus on being important to include in PRHISM were adapted where necessary and presented to participants in round 3 following a method similar to Mete et al [38]. Principles were adapted to make them relevant to social media and incorporate participant comments that were agreed upon in round 2. Similar principles where consensus was reached in round 2 were merged, as the 4 original tools have comparable aims and applications and, hence, some similar items. The adaptation and merging of principles were initially performed by the lead author (ED). Once updated, principles were circulated to the authors, and disagreements about the changes were discussed until an agreement was reached. Several principles were deemed appropriate and did not need to be amended. These were omitted from round 3 as consensus had already been reached in round 2.

A summary of how the principles had been adapted and merged was provided at the start of round 3. Participants were asked to rate their level of support for the inclusion of the adapted principles in the final PRHISM tool on a 5-point Likert scale ranging from *strongly oppose* to *strongly support*. Principles that did not achieve consensus in round 2 were also included in round 3. Participants were presented with feedback in the same format as in round 2 and asked to consider the groups’ responses when rating the importance of these items again.

### Consensus

Consensus was calculated after rounds 2 and 3 for each survey item. It was deemed that consensus had been met if ≥80% of participants selected 4 or 5 (important) or 1 or 2 (not important) on the Likert scale. Although determining consensus varies in Delphi methods [34,35,40], the 80% cutoff point was chosen as it has been suggested as an appropriate figure in some of the Delphi literature [40] and has been used in a similar Delphi study [38]. Stability of responses between survey iterations is often assessed in Delphi studies to aid in determining whether a consensus has been achieved [40,41]. There is a limited agreement in the literature on how to measure the stability of participant responses, and increasing weighted κ values is the most consistently advised method [40,41]. Owing to the adaptation of the principles included in the iterative surveys, it was not possible to calculate the κ values, and no measure of response stability was included. This approach is consistent with a recent Delphi study with aims and methods similar to this study [38].

### Development of PRHISM and Scoring Tool

Principles that reached a consensus were included in the final PRHISM tool. If consensus was not achieved by round 3, it was concluded that the principle was not relevant to be included in PRHISM. For those principles where consensus emerged only in round 3 (and not in previous rounds), the process of adapting and merging the principles was repeated. Once agreement about the adaptation of principles had been reached among the authors, the updated principles were also included in PRHISM.

A scoring system with instructions was also developed, which outlines the criteria to define what can be considered low-, moderate-, or high-quality information. This was based on the handbook and scoring system of the DISCERN instrument, which uses a 5-point Likert scale for rating the degree to which each quality principle has been met [29]. Participant comments where consensus was reached in round 2 were also used to inform the instructions for how each principle should be scored when evaluating health-related information on social media. After the draft of the PRHISM instructions and scoring tool had been finalized, participants who had completed at least round 1 were invited to provide final feedback. Although not an official survey round, this step enabled communication of the agreed principles and captured any final expert perspectives on the applications and utility of the tool.

## Results

### Overview

A total of 34 eligible experts consented to participate in this study, of whom 26 (76%) completed round 1, a total of 23 (68%) completed round 2, and 18 (53%) participants completed round 3 (69% retention rate from round 1). Participants were from Australia, Italy, China, New Zealand, the United Kingdom, the United States, and Vietnam and had expertise in a range of health-related disciplines (Table 1). Participants who completed all 3 Delphi rounds had an average of 10 (SD 8) years of experience in their health-related discipline, with a minimum of 3 and a maximum of 21 years of experience. The participants used a range of social media platforms (Table 1). Holding a personal social media account for professional purposes, managing the social media account of a health-related professional organization, and creating blog content were reported as participants’ professional health-related uses of social media.

**Table 1:** Participant characteristics across 3 rounds of the Delphi process (N=26).

Characteristics			Round 1 (n=26), n (%)		Round 2 (n=23), n (%)		Round 3 (n=18), n (%)
**Eligibility criteria met^a^**							
	Researcher or academic	15 (58)		14 (61)		12 (67)	
	Health professional	10 (38)		8 (35)		5 (28)	
	Communications or social media staff	4 (15)		4 (17)		3 (17)	
**Health-related field^a^**							
	Allied health	9 (35)		7 (30)		5 (28)	
	Public health	10 (38)		9 (39)		8 (44)	
	Medicine and clinical care	4 (15)		3 (13)		2 (11)	
	Preventive health	14 (54)		14 (61)		11 (61)	
**Highest level of education**							
	Doctorate	10 (38)		9 (39)		7 (39)	
	Master’s degree	10 (38)		9 (39)		8 (44)	
	Graduate diploma	1 (4)		—^b^		—	
	Bachelor’s degree with Honors	2 (8)		2 (9)		1 (6)	
	Bachelor’s degree	3 (12)		3 (13)		2 (11)	
**Location**							
	Australia	20 (77)		17 (74)		13 (72)	
	Other^c^	6 (23)		6 (26)		5 (28)	
**Social media use^a^**							
	Personal account for professional use	24 (92)		21 (91)		17 (94)	
	Management of professional organization’s account	12 (46)		10 (43)		7 (39)	
	Blog writing	5 (19)		4 (17)		2 (11)	
**Social media platforms used^a^**							
	Facebook	19 (73)		17 (74)		13 (72)	
	LinkedIn	21 (81)		18 (78)		15 (83)	
	Snapchat	3 (12)		3 (13)		2 (11)	
	Instagram	18 (69)		16 (70)		11 (61)	
	YouTube	9 (35)		8 (35)		6 (33)	
	Twitter	24 (92)		21 (91)		17 (94)	
	Pinterest	1 (4)		1 (4)		1 (6)	
	Reddit	2 (8)		1 (4)		1 (6)	
	TikTok	2 (8)		1 (4)		1 (6)	
	Clubhouse	1 (4)		—		—	

^a^Participants could fall under >1 category or select >1 option.

^b^Not available.

^c^One participant from Italy, China, New Zealand, United States, United Kingdom, and Vietnam each.

### Round 1

**Figure 1:**
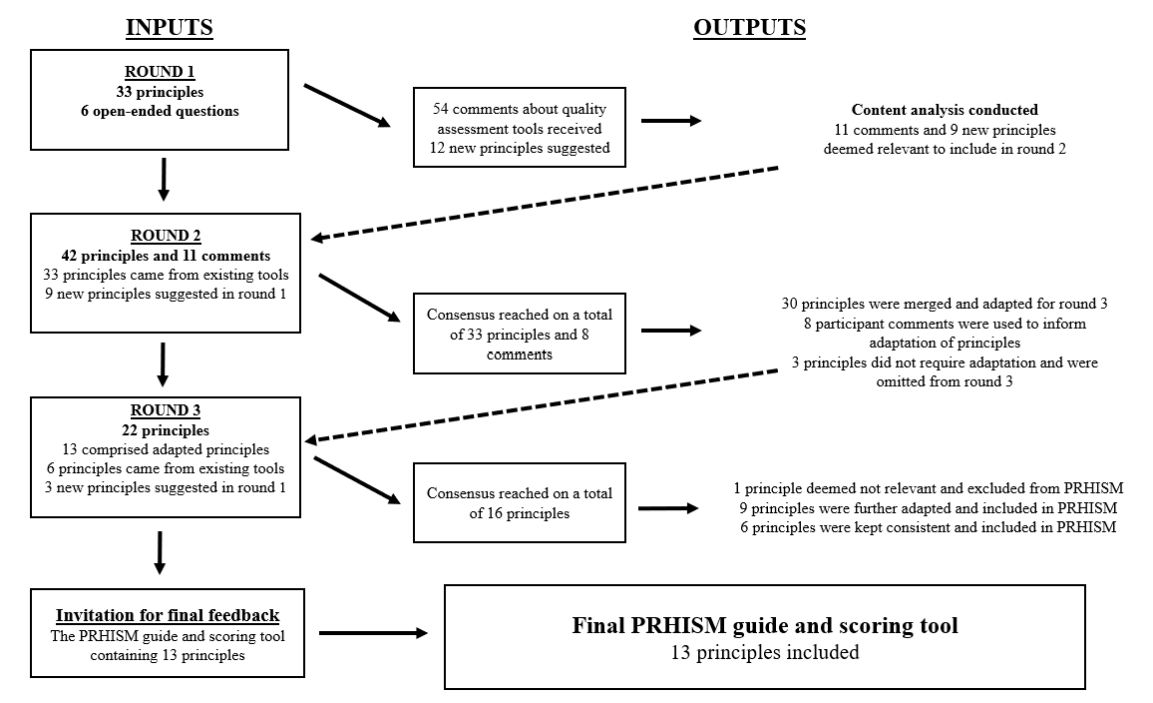
Flowchart of Delphi process adapted from Mete et al [38]. PRHISM: Principles for Health-related Information on Social Media.

The Delphi process is summarized in Figure 1 [38]. Participants rated a total of 33 items from 4 pre-existing quality assessment tools and were presented with 6 optional open-ended questions. A total of 54 comments were received in response to the open-ended questions in round 1. Participants also suggested 12 new principles that they considered important to the provision of quality health-related information on social media and that had not already been outlined in the quality assessment tools presented to them. After the content analysis was performed on the open-ended responses from round 1, a total of 11 comments about existing principles and 9 new principles were carried forward to round 2.

### Round 2

A total of 6 new principles, 27 principles from existing tools, and 8 participant comments reached consensus in round 2. All principles reaching consensus achieved consensus with ≥80% of participants selecting 4 or 5 on the Likert scale (ie, important), and none of the principles achieved consensus for participants selecting 1 or 2 (ie, unimportant). Similarly, all comments with consensus had ≥80% of participants selecting 4 or 5 on the Likert scale (ie, agree). Of the 33 principles that reached consensus, 30 (91%) were updated to incorporate consensus comments and merged where similar to form 13 adapted principles, which were included in round 3. The 3 remaining principles with consensus did not require updating and were thus omitted from round 3. The consensus items from round 2 and their adaptations are summarized in Multimedia Appendix 1.

### Round 3 and Development of PRHISM

Consensus was reached on a total of 16 principles. Of these 16 principles, 10 (62%) were adapted principles, 4 (25%) were principles from existing quality assessment tools, and 2 (13%) were new principles suggested in round 1. Similar to round 2, all principles with consensus in round 3 achieved ≥80% agreement through participants selecting 4 or 5 on the Likert scale (ie, important). One of the items from an existing tool reached consensus in round 3 (83% agreement) but was not included in PRHISM (“Does the information achieve its aims?” DISCERN instrument, question 2) [29]. It was excluded as it depended upon an adapted principle that did not achieve consensus (78% agreement) in round 3 (“The aims of health-related information provided on social media should be clearly outlined.” Adapted from the DISCERN instrument, question 1). The research team excluded both items from PRHISM as the importance of stating and achieving aims was only mentioned by 1 of the 4 tools in the surveys. Therefore, it was less likely to be critical to the provision of quality health-related information.

Of the remaining 15 principles that achieved consensus in round 3, a total of 6 (40%) were kept consistent and included in PRHISM, and 9 (60%) were further merged and adapted before being included in PRHISM, including 1 principle, which was merged with a consensus principle from round 2 that was not tested in round 3. The additional 2 principles that achieved consensus in round 2, and thus not tested in round 3, were also included, providing a total of 13 principles in PRHISM (Textbox 1). These 13 principles outline the gold standard for high-quality health-related social media content. The consensus items from round 3 and their adaptations are summarized in Multimedia Appendix 1.

**Final principles of the Principles for Health-related Information on Social Media tool.**

**Principle and description**

Principle 1: authorship

When providing health-related information on social media, the authors and contributors, their credentials, and their affiliations should be clearly stated on the social media profile. If this information cannot fit on a profile, crediting an authoritative institution is sufficient, if relevant. If not, all the contributors, their credentials, and their affiliations should be included.

Principle 2: authoritative

Health-related information provided on social media should be provided by qualified professionals, including health and medical scientists, and information should be within the scope of practice of the author’s qualifications. If information is provided by an unqualified person, this should be clearly indicated.

Principle 3: action oriented

Health-related information provided on social media should be action oriented and include clear, succinct messages to support decision-making and provide context for the consumer.

Principle 4: financial disclosure

Sponsorship, advertising, funding arrangements, and financial support or any potential conflicts of interest should be fully disclosed in a prominent and clear manner. Financial support and conflicts of interest can be disclosed on the social media profile. However, if a post has been sponsored, paid for, and contains advertising or a product that has been gifted, this needs to be clearly and prominently disclosed in the social media post.

Principle 5: attribution

Health-related information on social media should include clear references and hyperlinks to the original source of information used to compile the post. It should be clear when the original source of information was published. If all references cannot fit into the social media post, a link to the references and further information should be provided.

Principle 6: balance and justifiability

Health-related information provided on social media, which includes claims relating to the benefits or performance of a particular treatment, product, service, or behavior, should be balanced, unbiased, and supported by appropriate and quality evidence. The use of causative language and “shock tactics” should be avoided, and information about limitations or contrasting findings should be included.

Principle 7: risks and benefits

Health-related information provided on social media about a particular treatment, product, service, or behavior should clearly outline associated risks and benefits.

Principle 8: privacy

Health-related information on social media should respect principles of privacy and confidentiality. For example, if information, images, or videos of or about others are shared, they should be shared with permission.

Principle 9: complementary information

Health-related information provided on social media should provide support for individuals’ relationships with their physicians and other professional health care providers and should not be designed to replace such relationships. Support for discussion of options with the individuals’ health care provider should be included in posts containing health-related information.

Principle 10: referrals and support

Health-related information provided on social media should include referrals to additional sources of support and information. Where possible, links to such resources should be included.

Principle 11: readability and comprehensibility

Health-related information on social media should avoid the use of technical language and medical jargon. Plain language should be used, and information should be easily understandable by the general public and written at a grade 5 reading level.

Principle 12: accessibility

Medical and health information provided on social media should be accessible to individuals with vision and hearing impairments. For example, where relevant, social media posts that include images should provide alternative text in the caption, and videos should include closed captions.

Principle 13: images

Images included in health-related social media posts should be visually appealing and reflect rather than contradict the information provided in the post.

### Final Comment Stage

A total of 13 principles and instructions on the operationalization and scoring of each principle was sent to 26 participants. One of the participants responded with minor feedback and questions. No objections to the wording of the principles or instructions were received, and the principles were not adapted any further. The final PRHISM Guide and Scoring Tool is provided in Multimedia Appendix 2 [39,42-48].

## Discussion

### Principal Findings

This study used a Delphi approach to develop PRHISM. A total of 13 principles were established to define high-quality information and evaluate the quality of health-related social media content. PRHISM can be used to evaluate health-related social media posts intended for nonspecialist audiences, which are posted by public accounts on any social media platform or blog, and can be used as a research tool or to inform the development and selection of high-quality content. Many of the principles included in PRHISM are similar to principles and concepts from other tools [28-31,39]. However, their content and operationalization have been adapted to suit social media. The items in PRHISM can be broadly categorized into 4 themes: accessibility, transparency, authoritative and evidence-based information, and complementary relationships between patients and health professionals. These principles were agreed upon by experts recruited into the Delphi process and are well supported by the literature.

### PRHISM Themes

Accessibility is covered in principles 3, 11, and 12 of PRHISM. Principle 3 stipulates that health-related information should be action oriented, clear, succinct, and facilitative of decision-making. This is in line with evidence that recommends providing practical and simple health-related information that can be easily implemented [38,49]. Improving accessibility by providing health-related information in readable and plain language is outlined in principle 11. It is widely agreed that written health-related information should not be above an eighth-grade reading level [50,51], and for greater inclusivity, no higher than fifth grade has been suggested [52]. Generally, readability has not been included in widely used quality assessment tools, although it has often been evaluated alongside information quality in health research [27]. The inclusion of principle 11 in PRHISM is supported by literature that emphasizes the importance of providing written and nonwritten health-related information in simple and plain language [42,52], and recent research indicates that this is also pertinent to social media [53]. For the information to be accessible, it should be easily understood the first time it is heard or read [42].

Principle 12 specifies that health-related social media content should be accessible to individuals with vision and hearing impairments. Those living with vision and hearing impairments typically have poor health literacy and face challenges when accessing health-related information [54]. There have been recent calls to address this issue and provide guidance to health professionals to deliver accessible information [54]. Interestingly, principles 3, 11, and 12, which relate to various components of accessibility, were new principles suggested by the participants, and such considerations have not been included in previous tools [28-31,39]. This may reflect a greater understanding among experts regarding the importance of health-related information that meets the needs of all members of the population. The need for accessible health-related information has been advocated in the literature and supports the inclusion of principles related to accessibility in PRHISM [38,42,49-52,54]. If adhered to, principles 3, 11, and 12 may assist consumers with diverse needs and improve their overall accessibility to health advice provided on social media.

Of the 13 principles included in PRHISM, 7 (54%) pertained to transparency. Principles 1 and 5 state the need for authors to specify their credentials and qualifications and provide details about the original sources of information used to compile the social media content, respectively. The components of information quality outlined in principles 1 and 5 were covered in all the tools included in the Delphi surveys [28-31], as well as several other commonly used quality assessment tools [27], indicating their importance. Providing financial and conflict of interest disclosures is specified in principle 4 of PRHISM and received very strong support from participants (34/34, 100% rated as important or very important). As social media marketing and *influencing* has expanded, advertising associations have released statements declaring the need for prominent and clear disclosures of advertisements and other conflicts of interest in social media content [43,44]. The importance of providing such disclosures in health-related social media content has also been echoed in recent literature [53], and failure to disclose relevant conflicts of interest has the power to erode the public’s trust in authoritative voices [55].

Transparency through the provision of comprehensive and balanced information is stipulated under principles 6 and 7 of PRHISM. These principles state that health-related claims should provide complete information on risks and benefits and clearly outline the limitations or areas of uncertainty. Comprehensive and balanced information provision is fundamental to the DISCERN instrument [28] and QUEST [39]. Furthermore, disclosing all relevant contexts and limitations in health-related social media content has been outlined as necessary to ensure that consumers can reach informed conclusions [53]. Others have acknowledged that although transparency in health communication is important, it is complex and, in some instances, may work against public understanding [56]. For example, research has shown that transparent information on scientific uncertainties and the risks and benefits of certain health behaviors has led to consumer confusion, which can undermine the public’s trust in science [56,57]. Transparency is emphasized throughout all prominent quality assessment tools [27-31] and has been highlighted as critical to the credibility of health-related information sources on social media [53,58], supporting its centrality to most of the items included in PRHISM. This creates a challenge for content creators in balancing elements of transparency and accessibility to ensure that information is complete, credible, and understandable.

The need for quality health-related information on social media to be evidence based and authoritative is covered in principles 2 and 6 of PRHISM. Such considerations are not new and have long been recognized as fundamental to the provision of high-quality science and health-related information [27]. Recently, authoritative sources and evidence-based information have been identified as key credibility attributes for health-related social media content [53,58]. Others have highlighted the need for propagation of evidence-based information on social media to curtail the influence of misinformation on health issues such as vaccine hesitancy [59] and have also noted the responsibility of authoritative voices to combat and refute sources of misinformation on social media [60].

Finally, principles 9 and 10 are centered on supporting the relationships between patients and their health care providers. The DISCERN instrument, HONcode principles, and QUEST all express the importance of health-related information that is complementary to advice from health professionals [28,29,31,39]. Although social media can be a useful and powerful tool for the dissemination of health-related information [60], most information is not personalized and does not consider individuals’ medical history or health needs. Codes of conduct apply to registered health care providers, and the provision of personalized health advice via social media is considered unethical and has resulted in the formal investigation of medical professionals [61,62]. Principles 9 and 10 accord with the health information–seeking behavior literature, which acknowledges that although web-based information environments can be interactive, they are not sufficient to replace the tailored care and advice that professionals can provide in a health care setting [6,63]. Research has shown that discussing web-based health-related information with a relevant professional can help strengthen patient-physician relationships and bolster shared decision-making [63,64].

### Strengths and Limitations

This study had a number of strengths. First, the Delphi technique was a highly appropriate method for meeting the aim of this research, and the sample size was suitable to produce meaningful results [35,40]. Second, the retention rate (69% retention of round 1 participants) was high, particularly given the generally low retention rates observed in Delphi studies [35,40]. Third, most of the principles included in PRHISM were adapted from tools that are widely used and have been shown to have good reliability [27-31,39]. Fourth, PRHISM is suitable for the evaluation of information from all health-related disciplines, and the accompanying scoring tool and guide for use will assist researchers when using the tool. Finally, PRHISM can also be used by health professionals to guide the development of quality health-related social media content and inform consumers about the attributes of quality information.

This investigation also had a number of limitations. First, academics, researchers, and respondents from Australia were overrepresented in the study sample, which may have limited the range of the perspectives captured. In addition, a general limitation of the Delphi method is that consensus does not necessarily mean that the correct answer has been chosen [35], which was evident in this study when 2 opposing principles remained after round 3. The research team conferred and discussed the face validity of these principles, informed by the literature and the overall Delphi process to mitigate the impact, to form a final decision. Fourth, principle 13 focused on image-based content and did not mention video content. This principle may have been more comprehensive if it also focused on video content; however, this was not considered until after the principles and PRHISM guide had been sent to participants for final comment. Finally, although PRHISM has been designed to be flexible to the changing social media landscape, the tool may require updates as social media continues to evolve.

### Implications and Future Directions

PRHISM may contribute to measuring and improving the quality of health-related information on social media. However, other strategies will also be required. Greater efforts by social media platforms to prevent the propagation of misinformation and direct consumers to credible information are needed. Some social media companies have taken steps to remove or provide warnings about health-related misinformation on their platforms [65,66]. Nevertheless, their impact is likely insufficient, and greater regulation, moderation, and fact checking by social media platforms are needed [53,59]. A practical implication of PRHISM is that the principles may be used to inform such regulation of social media platforms to make high-quality health information more prominent and limit the propagation of low-quality information. Greater efforts are also required to provide education to improve consumers’ health and media literacy so that they are better able to identify credible health-related information from dangerous misinformation. A further practical implication of this study is that the principles may form the basis of educational materials to help develop health and media literacy. Finally, PRHISM is the first quality assessment tool for health information specific to social media and provides a standardized measure of information quality. Future studies that aim to assess the quality of health-related social media content should use PRHISM, instead of quality assessment tools that are not specific to social media, to improve the measurement of health information in social media research.

### Conclusions

Previously developed quality assessment tools for evaluating health-related information are not appropriate for evaluating social media content as they do not consider the unique characteristics of social media, and this study served to address this gap. Resulting from a comprehensive Delphi process, PRHISM comprised 13 principles that can be used by researchers to evaluate the quality of health-related information on social media. The principles promote accessibility, transparency, and authoritative and evidence-based information provision, supporting relationships between consumers and health care providers. The information contained in the PRHISM guide (Multimedia Appendix 2) defines what can be considered low-, moderate-, and high-quality information and sets out instructions for use in a research setting. Information from the PRHISM guide can also be used by health professionals and content creators to inform the provision of high-quality health-related social media content and by consumers to help identify high- and low-quality information. Further research is needed to improve the media and health literacy skills of the general population and regulate misleading poor-quality health-related information on social media platforms to reduce the threat of misinformation to public health.

## Appendix

Multimedia Appendix 1 Supplementary tables - Principles for Health-related Information on Social Media Delphi Round 2 and 3 consensus items and adaptations.

Multimedia Appendix 2 Principles for Health-related Information on Social Media (PRHISM) guide and scoring tool.

## Abbreviations

HONcode: Health on the Net
JAMA: Journal of the American Medical Association
PRHISM: Principles for Health-related Information on Social Media
QUEST: Quality Evaluation Scoring Tool
