# Supplementary material for: Development of Principles for Health-Related Information on Social Media: Delphi Study
Source: J Med Internet Res. 2022 Sep 8;24(9):e37337. doi: 10.2196/37337 (PMC9501680; doi:10.2196/37337)
Supplement: Multimedia Appendix 1 [file jmir_v24i9e37337_app1.docx]

Supplementary Table 1: PRinciples for Health-related Information on Social Media (PRHISM) Delphi Round 2 consensus items and adaptations

| **Item included in Round 2** | **Agreement (%)** | **Merged/adapted principle included in Round 3** |
| --- | --- | --- |
| Authorship: Authors and contributors, their affiliations, and relevant credentials should be provided. (JAMA 1) | 91 | Authorship:  When providing health-related information on social media, the authors and contributors, their credentials and affiliations should be clearly stated on the social media profile. If this information cannot fit on a profile, crediting an authoritative institution is sufficient, if relevant. If not, all the contributors, their credentials and affiliations should be included. |
| It may not always be possible to include all contributors and their affiliations and credentials within a social media post. Crediting an authoritative institution (such as a university or professional/heath organisation) is sufficient, if relevant. If not, the contributor, their affiliations and credentials should be included. (Participant comment about JAMA 1) | 100 |  |
| Authorship:  0 - No indication of authorship or username  1 - All other indications of authorship  2 - Author's name and qualification clearly stated (QUEST 1) | 91 |  |
| Authoritative: Any medical or health advice provided and hosted on this site will only be given by medically trained and qualified professionals unless a clear statement is made that a piece of advice offered is from a non-medically qualified individual or organisation. (HONCode 1) | 96 | Authoritative:  Health-related information provided on social media should be given by qualified professionals, including health and medical scientists, and information should be within the scope of practice of the author’s qualifications. If information is provided by an unqualified person, this should be clearly indicated. |
| Health and medical scientists should be included as authoritative persons. (Participant comment about HONCode 1) | 87 |  |
| The author's qualifications should be listed, and the health information provided should be within the scope of practice for these qualifications. (Comment about HONCode 1) | 100 |  |
| Attribution: References and sources for all content should be listed clearly, and all relevant copyright information noted. (JAMA 2) | 83 | Attribution:  Health-related information on social media should include clear references and hyperlinks to the original source(s) of information used to compile the post. It should be clear when the original source of information was published. If all references cannot fit into the social media post, a link to the references and further information should be provided. |
| If references cannot fit into a social media post a link to the references and further information should be provided. (Comment about JAMA 2) | 91 |  |
| Attribution: Where appropriate, information contained on this site will be supported by clear references to source data and, where possible, have specific HTML links to that data. The date when a clinical page was last modified will be clearly displayed (e.g. at the bottom of the page). (HONCode 4) | 91 |  |
| Is it clear what sources of information were used to compile the publication (other than the author)? (DISCERN 4) | 91 |  |
| Is it clear when the information used or reported in the publication was produced? (DISCERN 5) | 83 |  |
| Attribution:  0 - No sources  1 - Mention of expert source, research findings (though with insufficient information to identify the specific studies), links to various sites, advocacy body, or other  2 - Reference to at least one identifiable scientific study, regardless of format (e.g., information in text, reference list)  3 - Reference to mainly identifiable scientific studies, regardless of format (in >50% of claims) (QUEST 2) | 96 |  |
| Action-orientated:  Health-related information provided on social media should be action-orientated and include clear, succinct messages to support decision making and provide context for the consumer. | 96 | * |
| Financial disclosure: Support for this website will be clearly identified, including the identities of commercial and non-commercial organisations that have contributed funding, services or material for the site. (HONCode 7) | 96 | Financial disclosure:  Sponsorship, advertising, funding arrangements, financial support or any potential conflicts of interest should be fully disclosed in a prominent and clear manner. Financial support and conflicts of interest can be disclosed on the social media profile. However, if a post has been sponsored, paid for, contains advertising and/or a product that has been gifted this needs to be clearly and prominently disclosed in the social media post. |
| Disclosure: Website "ownership" should be prominently and fully disclosed, as should any sponsorship, advertising, underwriting, commercial funding arrangements or support or potential conflicts of interest. This includes arrangements in which links to other sites are posted as a result of financial considerations. (JAMA 3) | 91 |  |
| It is important to disclose conflicts of interest so that readers can critically evaluate the information provided. Conflicts of interest can be disclosed on the social media profile, rather than within individual posts. However, in the instance of posts that have been paid for (i.e. promoted posts) this should be clearly disclosed within the post. (Comment about JAMA 3) | 100 |  |
| Advertising: If advertising is a source of funding it will be clearly stated. A brief description of the advertising policy adopted by the website owners will be displayed on the site. Advertising and other promotional material will be presented to the viewers in a manner and context that facilitates differentiation between it and the original material created by the institution operating the site. (HONCode 8) | 96 |  |
| Relationships concerning financial disclosure and advertising of health professionals needs to be very transparent. There are guidelines around acknowledging sponsoring or funding for posting certain content on social media, and these should be adhered to. (Comment about HONCode 8) | 96 |  |
| Conflict of interest:  0 - Endorsement or promotion of intervention designed to prevent or treat condition (e.g., supplements,  brain training games, foods) within the article  1 - Endorsement or promotion of educational products and services (e.g., books, care home service)  2 - Unbiased information (QUEST 3) | 96 |  |
| Complementary: The information provided on this site is designed to support, not replace, the relationship that exists between a patient/site visitor and their existing physician. (HONCode 2) | 96 | Complementary:  Health-related information provided on social media should provide support for individuals’ relationships with their doctor and other professional healthcare providers and should not be designed to replace such relationships. Support for discussion of options with the individuals’ healthcare provider should be included in posts containing health-related information. |
| "Does the information provide support for shared decision-making?" Support for shared decision-making includes encouragement and support for the patient to discuss treatment options with their healthcare provider, partner, friends and/or family. (DISCERN 15) | 87 |  |
| Complementarity:  0 - No support of the patient-physician relationship  1 - Support of the patient-physician relationship (QUEST 5) | 87 |  |
| Justifiability: Any claims relating to the benefits/performance of a specific treatment, commercial product or service will be supported by appropriate, balanced evidence in the manner outlined above in Principle 4. (HONCode 5) | 96 | Balance & justifiability:  Health-related information provided on social media that includes claims relating to the benefits/performance of a particular treatment, product, service or behaviour should be balanced, unbiased and supported by appropriate and quality evidence. The use of causative language and ‘shock tactics’ should be avoided and information about limitations and/or contrasting findings included. |
| Is it balanced and unbiased? (DISCERN 6) | 100 |  |
| Is it clear that there may be more than one possible treatment choice?(DISCERN 14) | 91 |  |
| Information outlining the level and quality of available evidence should be included in health-related social media posts. (New principle) |  |  |
| Tone (includes title):  0 - Fully supported (authors fully and unequivocally support the claims, strong vocabulary such as "cure", "guarantee", and "easy", mostly use of non-conditional verb tenses ("can", "will"), no discussion of limitations)  1 - Mainly supported (authors mainly support their claims but with more cautious vocabulary such as "can reduce your risk" or "may help prevent", no discussion of limitations)  2 - Balanced/cautious support (authors' claims are balanced by caution, includes statements of limitations and/or contrasting findings) (QUEST 6) | 100 |  |
| Is the information relevant? (DISCERN 3) | 100 | Relevance:  Health-related information provided on social media should be relevant to the intended target group and the target group should be identified. |
| Social media messages need to identify the target group and who the information is relevant for. (New principle) |  |  |
| Currency: Dates that content was posted and uploaded should be indicated. (JAMA 4) | 91 | Currency:  The date that health-related information was posted on social media should be indicated. |
| Currency:  0 - No date present  1 - Article is dated but 5 years or older  2 - Article is dated within the last 5 years (QUEST 4) | 87 |  |
| Does it describe the benefits of each treatment? (DISCERN 10) | 91 | Risks & benefits:  Health-related information provided on social media about a particular treatment, product, service or behaviour should clearly outline associated risks and benefits. |
| Does the information describe the risks of each treatment? (DISCERN 11) | 96 |  |
| Statements 10 and 11 go together. If it described the benefits, it must describe the risks. (Comment about DISCERN 10 & 11) | 91 |  |
| Privacy: Confidentiality of data relating to individual patients and visitors to a medical/health website, including their identity, is respected by this website. The website owners undertake to honour and exceed the legal requirements of medical/health information privacy that apply in the country and state where the website and mirror sites are located. (HONCode 3) | 83 | Privacy:  Health-related information on social media should respect principles of privacy and confidentiality. For example, if information, images or videos of or about others are shared, they are shared with permission. |
| Are the aims of the information clear? (DISCERN 1) | 96 | Aims  The aims of health-related information provided on social media should be clearly outlined. |
| Does the publication provide details of additional sources of support and information? (DISCERN 7) | 100 | Referrals & support:  Health-related information provided on social media should include referrals to additional sources of support and information, where possible, links to such resources should be included. |
| Alternative text (a.k.a. 'alt text'*) should be provided when a post includes images to ensure that health information is accessible to low vision/blind persons to increase accessibility for disabled persons. *Alt text provides a description of the appearance and/or function of an image on a page. (New principle) |  | Accessibility:  Medical and health information provided on social media should be accessible to vision- and hearing-impaired individuals. For example, where relevant, social media posts that include images should provide alternative text* in the caption and videos should include closed captions.  *Alternative text, also known as ‘alt text’ provided a description of the appearance and/or function of an image. |
| Readability  Health-related information on social media should avoid the use of technical language and medical jargon. Plain language should be used, information should be easily understandable by the general public and written at a Grade 5 reading level. (New principle) |  | * |
| Images  Images included in health-related social media posts should reflect the information provided and not contradict the information included in the post. (New principle) |  | * |

* Principle did not require adaptation after Round 2

Supplementary Table 2: PRinciples for Health-related Information on Social Media (PRHISM) Delphi Round 3 consensus items and adaptations

| **Item from Round 3** | **Agreement (%)** | **Merged/adapted principle included in PRHISM** |
| --- | --- | --- |
| Images included in health-related social media posts should reflect the information provided and not contradict the information included in the post. | 100* | Images:  Images included in health-related social media posts should be visually appealing and reflect rather than contradict the information provided in the post. |
| Health-related information on social media should be visually appealing. | 94 |  |
| Health-related information provided on social media that includes claims relating to the benefits/performance of a particular treatment, product, service or behaviour should be balanced, unbiased and supported by appropriate and quality evidence. The use of causative language and ‘shock tactics’ should be avoided and information about limitations and/or contrasting findings included. | 94 | **Balance & justifiability**:**  Health-related information provided on social media that includes claims relating to the benefits/performance of a particular treatment, product, service or behaviour should be balanced, unbiased and supported by appropriate and quality evidence. The use of causative language and ‘shock tactics’ should be avoided and information about limitations and/or contrasting findings included. |
| Does the information refer to areas of uncertainty? | 94 |  |
| Health-related information provided on social media about a particular treatment, product, service or behaviour should clearly outline associated risks and benefits. | 89 | **Risks & benefits:  Health-related information provided on social media about a particular treatment, product, service or behaviour should clearly outline associated risks and benefits. |
| Does the information describe how treatment choices affect overall quality of life? | 89 |  |
| Health-related information on social media should include clear references and hyperlinks to the original source(s) of information used to compile the post. It should be clear when the original source of information was published. If all references cannot fit into the social media post, a link to the references and further information should be provided. | 89 | ** Attribution:  Health-related information on social media should include clear references and hyperlinks to the original source(s) of information used to compile the post. It should be clear when the original source of information was published. If all references cannot fit into the social media post, a link to the references and further information should be provided. |
| Citations to peer-reviewed journal articles are included when claims state that content is based on research. | 83 |  |
| Health-related information provided on social media should include referrals to additional sources of support and information, where possible, links to such resources should be included. | 94 | Referrals & support:  Health-related information provided on social media should include the author’s contact information and referrals to additional sources of support and information. Where possible, links to such resources should be included. |
| The designers of this website will seek to provide information in the clearest possible manner and provide contact addresses for visitors that seek further information or support. The webmaster (website owner) will display their email address clearly throughout the website. | 83 |  |
| Authorship:  When providing health-related information on social media, the authors and contributors, their credentials and affiliations should be clearly stated on the social media profile. If this information cannot fit on a profile, crediting an authoritative institution is sufficient, if relevant. If not, all the contributors, their credentials and affiliations should be included. | 94 | *** Authorship:  When providing health-related information on social media, the authors and contributors, their credentials and affiliations should be clearly stated on the social media profile. If this information cannot fit on a profile, crediting an authoritative institution is sufficient, if relevant. If not, all the contributors, their credentials and affiliations should be included. |
| Authoritative:  Health-related information provided on social media should be given by qualified professionals, including health and medical scientists, and information should be within the scope of practice of the author’s qualifications. If information is provided by an unqualified person, this should be clearly indicated. | 94 | *** Authoritative:  Health-related information provided on social media should be given by qualified professionals, including health and medical scientists, and information should be within the scope of practice of the author’s qualifications. If information is provided by an unqualified person, this should be clearly indicated. |
| Financial disclosure:  Sponsorship, advertising, funding arrangements, financial support or any potential conflicts of interest should be fully disclosed in a prominent and clear manner. Financial support and conflicts of interest can be disclosed on the social media profile. However, if a post has been sponsored, paid for, contains advertising and/or a product that has been gifted this needs to be clearly and prominently disclosed in the social media post. | 100 | *** Financial disclosure:  Sponsorship, advertising, funding arrangements, financial support or any potential conflicts of interest should be fully disclosed in a prominent and clear manner. Financial support and conflicts of interest can be disclosed on the social media profile. However, if a post has been sponsored, paid for, contains advertising and/or a product that has been gifted this needs to be clearly and prominently disclosed in the social media post. |
| Complementary:  Health-related information provided on social media should provide support for individuals’ relationships with their doctor and other professional healthcare providers and should not be designed to replace such relationships. Support for discussion of options with the individuals’ healthcare provider should be included in posts containing health-related information. | 83 | *** Complementary:  Health-related information provided on social media should provide support for individuals’ relationships with their doctor and other professional healthcare providers and should not be designed to replace such relationships. Support for discussion of options with the individuals’ healthcare provider should be included in posts containing health-related information. |
| Accessibility:  Medical and health information provided on social media should be accessible to vision- and hearing-impaired individuals. For example, where relevant, social media posts that include images should provide alternative text* in the caption and videos should include closed captions.  *Alternative text, also known as ‘alt text’ provided a description of the appearance and/or function of an image. | 89 | *** Accessibility:  Medical and health information provided on social media should be accessible to vision- and hearing-impaired individuals. For example, where relevant, social media posts that include images should provide alternative text^1^ in the caption and videos should include closed captions.  ^1^Alternative text, also known as ‘alt text’ provided a description of the appearance and/or function of an image. |
| Readability:  Health-related information on social media should avoid the use of technical language and medical jargon. Plain language should be used, information should be easily understandable by the general public and written at a Grade 5 reading level. | * | ***Readability:  Health-related information on social media should avoid the use of technical language and medical jargon. Plain language should be used, information should be easily understandable by the general public and written at a Grade 5 reading level. |
| Privacy:  Health-related information on social media should respect principles of privacy and confidentiality. For example, if information, images or videos of or about others are shared, they are shared with permission. | 94 | ***Privacy:  Health-related information on social media should respect principles of privacy and confidentiality. For example, if information, images or videos of or about others are shared, they are shared with permission. |
| Action-orientated:  Health-related information provided on social media should be action-orientated and include clear, succinct messages to support decision making and provide context for the consumer. | * | ***Action-orientated:  Health-related information provided on social media should be action-orientated and include clear, succinct messages to support decision making and provide context for the consumer. |

*Consensus reached in Round 2 and item not included in Round 3, percent agreement is from Round 2.

**Wording of principle was not adapted but instructions in PRHISM Guide incorporated additional consensus items

*** Principle did not require adaptation after Round 3
