# Supplementary material for: Development of Principles for Health-Related Information on Social Media: Delphi Study
Source: J Med Internet Res. 2022 Sep 8;24(9):e37337. doi: 10.2196/37337 (PMC9501680; doi:10.2196/37337)
Supplement: Multimedia Appendix 2 [file jmir_v24i9e37337_app2.docx]

**The PRHISM Guide**

**PRinciples for Health Information on Social Media**

**Introduction**

PRinciples for Health Information on Social Media (PRHISM) was developed to provide standards for high quality health communication via social media. PRHISM is comprised of 13 principles and can be used to guide the development or evaluate the quality of health-related information provided on social media. The principles have been designed to be implemented in collaboration with the information provided in *The PRHISM Guide*.

**What is the scope of PHRISM?**

The *PHRISM Guide and Scoring Tool* have been designed to evaluate social media content about health that is intended for a non-expert audience. PRHISM is not appropriate for assessing social media content that contains health information intended for health professionals or health experts or information about public health policy. PHRISM can be used to evaluate publicly available social media posts that have been published by any author, including but not limited to health professionals, health experts, influencers and brands. The tool is appropriate for evaluating posts from any social media platform including blog content.

**Who is PRHISM for?**

*The PRHISM Guide* and *Scoring Tool* have been designed for use in research, policy and practice settings to evaluate social media content. *The PRHISM Guide* provides detailed information on how to determine a score for each principle and how to use the *Scoring Tool*.

**Using PRHISM to evaluate social media content**

Each of the 13 principles is scored on a five-point Likert scale ranging from ‘completely unmet’ (zero) to ‘completely met’ (four). When using PRHSIM to evaluate content, there are two scoring components that need to be completed for each principle, the *score* and the *possible score*:

- The *score* is the number from the Likert scale that has been awarded for the degree to which a principle has been met.
- The *possible score* is the highest possible number that could be awarded for each principle.

Some principles may not apply to all situations. When a principle is not applicable to the social media post being evaluated, a zero should be given for both *score* and *possible score*. When a principle is relevant to the social media post being analysed, the maximum score (4) should be assigned to the *possible score.*

Once each principle has been assessed, the numbers in the *score* column should be tallied together and the numbers in the *possible score* column should be tallied together. To determine the final PRHISM score for a social media post:

***Score/Possible score* X 100 = PRHISM Score**

The final PRHISM score can range from zero to 100. The closer to 100 the final score is, the greater the quality of the information.

**PRHISM Scoring Classification**

| **Score** | **Classification** |
| --- | --- |
| 0 - 25 | *Poor* |
| 26 - 50 | *Mediocre* |
| 51 - 75 | *Good* |
| 76 - 100 | *Excellent* |

**Before you start**

It is important to note that social media is constantly evolving and there are considerable differences between social media platforms. Where possible, a range of examples have been provided for each principle to provide context for the user. It is recommended that users familiarise themselves with the current characteristics of the social media platform(s) being researched before analysis begins. A transparent and repeatable search strategy will allow researchers to locate appropriate content to analyse. PRIHSM can be applied to analyse the content in each individual post.

**How was PRHISM developed?**

A modified Delphi approach was used to gain expert consensus on the principles and function of PRHISM. Health and social media experts were recruited and three surveys were administered between February and May 2021. The first survey was informed by a literature review and included open-ended questions and items from four existing quality assessment tools. Subsequent surveys were informed by the results to the proceeding survey. After the third survey principles were finalised and this instruction guide and scoring tool for PRHISM was developed and circulated to expert participants for final feedback. This study was funded and conducted as part of a PhD candidature at Deakin University, Melbourne, Australia.

**Suggested citation**

Denniss E, Lindberg R, McNaughton S. (2022). The Development of PRinciples for Health-related Information on Social Media (PRHISM): A Delphi study. Journal of Medical Internet Research. doi: https://www.jmir.org/2022/0/e0/

**PRHISM Scoring Tool**

| **Principle 1: Authorship**  *When providing health-related information on social media, the authors and contributors, their credentials, affiliations, and contact information should be clearly stated on the social media profile.* | | | | | | | | | | |
| --- | --- | --- | --- | --- | --- | --- | --- | --- | --- | --- |
| 0 | | 1 | | 2 | | 3 | | 4 | ***Score*** | ***Possible score*** |
| Completely unmet | | Partially met | | | | | | Completely met |  |  |
| **Principle 2: Authoritative**  *Health-related information provided on social media should be given by qualified professionals, including health and medical scientists, and information should be within the scope of practice of the author’s qualifications. If information is provided by an unqualified person, this should be clearly indicated.* | | | | | | | | | | |
| 0 | | 1 | | 2 | | 3 | | 4 | ***Score*** | ***Possible score*** |
| Completely unmet | | Partially met | | | | | | Completely met |  |  |
| **Principle 3: Action-orientated**  *Health-related information provided on social media should be action-orientated and include clear, succinct messages to support decision making and provide context for the consumer.* | | | | | | | | | | |
| 0 | | 1 | | 2 | | 3 | | 4 | ***Score*** | ***Possible score*** |
| Completely unmet | | Partially met | | | | | | Completely met |  |  |
| **Principle 4: Financial Disclosure**  *Sponsorship, advertising, funding arrangements, financial support or any potential conflicts of interest should be fully disclosed in a prominent and clear manner.* | | | | | | | | | | |
| 0 | | 1 | | 2 | | 3 | | 4 | ***Score*** | ***Possible score*** |
| Completely unmet | | Partially met | | | | | | Completely met |  |  |
| **Principle 5: Attribution**  *Health-related information on social media should include clear references and hyperlinks to the original source(s) of information used to compile the post.* | | | | | | | | | | |
| N/A | 0 | | 1 | | 2 | | 3 | 4 | ***Score*** | ***Possible score*** |
| N/A | Completely unmet | | Partially met | | | | | Completely met |  |  |
| **Principle 6: Balance & Justifiability**  *Health-related information provided on social media that includes claims relating to the benefits/performance of a particular treatment, product, service or behaviour should be balanced, unbiased and supported by appropriate and quality evidence.* | | | | | | | | | | |
| N/A | 0 | | 1 | | 2 | | 3 | 4 | ***Score*** | ***Possible score*** |
| N/A | Completely unmet | | Partially met | | | | | Completely met |  |  |
| **Principle 7: Risks & Benefits**  *Health-related information provided on social media about a particular treatment, product, service or behaviour should clearly outline associated risks and benefits.* | | | | | | | | | | |
| N/A | 0 | | 1 | | 2 | | 3 | 4 | ***Score*** | ***Possible score*** |
| N/A | Completely unmet | | Partially met | | | | | Completely met |  |  |
| **Principle 8: Privacy**  *Health-related information on social media should respect principles of privacy and confidentiality.* | | | | | | | | | | |
| N/A | 0 | | 1 | | 2 | | 3 | 4 | ***Score*** | ***Possible score*** |
| N/A | Completely unmet | | Partially met | | | | | Completely met |  |  |
| **Principle 9: Complementary Information**  *Health-related information provided on social media should provide support for individuals’ relationships with their doctor and other professional healthcare providers and should not be designed to replace such relationships. Support for discussion of options with the individuals’ healthcare provider should be included in posts containing health-related information.* | | | | | | | | | | |
| N/A | 0 | | 1 | | 2 | | 3 | 4 | ***Score*** | ***Possible score*** |
| N/A | Completely unmet | | Partially met | | | | | Completely met |  |  |
| **Principle 10: Referrals & Support**  *Health-related information provided on social media should include referrals to additional sources of support and information.* | | | | | | | | | | |
| 0 | | 1 | | 2 | | 3 | | 4 | ***Score*** | ***Possible score*** |
| Completely unmet | | Partially met | | | | | | Completely met |  |  |
| **Principle 11: Readability & Comprehensibility**  *Health-related information on social media should avoid the use of technical language and medical jargon. Plain language should be used and information should be easily understandable by the general public.* | | | | | | | | | | |
| 0 | | 1 | | 2 | | 3 | | 4 | ***Score*** | ***Possible score*** |
| Completely unmet | | Partially met | | | | | | Completely met |  |  |
| **Principle 12: Accessibility**  *Health-related information provided on social media should be accessible to vision- and hearing-impaired individuals.* | | | | | | | | | | |
| N/A | 0 | | 1 | | 2 | | 3 | 4 | ***Score*** | ***Possible score*** |
| N/A | Completely unmet | | Partially met | | | | | Completely met |  |  |
| **Principle 13: Images**  *Images included in health-related social media posts should be visually appealing and reflect rather than contradict the information provided in the post.* | | | | | | | | | | |
| N/A | 0 | | 1 | | 2 | | 3 | 4 | ***Score*** | ***Possible score*** |
| N/A | Completely unmet | | Partially met | | | | | Completely met |  |  |
| **Totals:** | | | | | | | | |  |  |
| ***Score/Possible score* X 100 = PRHISM Score**  **PRHISM Score:** | | | | | | | | |  | |

**Principle 1: Authorship**

*When providing health-related information on social media, the authors and contributors, their credentials, affiliations, and contact information should be clearly stated on the social media profile.*

**Scoring this principle**

Authorship information should be displayed on the social media profile, rather than in individual social media posts. When assessing Principle 1, the information provided in the author/account holder’s profile or bio should be evaluated.

Best practice inclusions:

- Account holders may be contactable via a social media platform’s direct message function. If available, this is sufficient contact information.
- It is common for account holders to close their direct messages on social media. In this instance, an alternative contact method such as an email address, should be supplied in the social media profile.
- Information about qualifications should include the level of qualification received. For example, it should be clear if the account holder holds a university qualifications and level of degree in the indicated health-related field.
- If all authorship information cannot fit on a profile, crediting an authoritative institution is sufficient, if relevant. If not, all the contributors, their credentials and affiliations should be included.
- If the social media account is held by a brand or company, the author of the health-related information provided should be identifiable on the post they have written or the social media profile. Their qualifications and credentials should be clearly provided.

| **Principle 1 Criteria** | **Score** |
| --- | --- |
| Comprehensive information about the account holder or author(s)’ identity, including their specific credentials and affiliations are included on the social media profile. Sufficient contact information is provided. | 4 |
| Identity of the account holder or author(s) is clear and mostly comprehensive information about the author(s)’ credentials and affiliations provided but information lacks clarity and/or key details are missing. Sufficient contact information is provided. | 3 |
| Identity of the account holder or author(s) is clear and some information about their credentials and affiliations are provided, however, the information lacks clarity and a number of details are missing. Contact information may or may not be sufficient. | 2 |
| Identity of the account holder or author(s) is clear, however, no information about credentials or affiliations provided and contact information is not sufficient. | 1 |
| No information about the identity or credentials of the account holder or author provided and contact information is not sufficient. | 0 |

**Principle 2: Authoritative**

*Health-related information provided on social media should be given by qualified professionals, including health and medical scientists, and information should be within the scope of practice of the author’s qualifications. If information is provided by an unqualified person, this should be clearly indicated.*

**Scoring this principle:**

Information about authorship (examined in Principle 1) from the account holder’s profile/bio and the information contained in the social media post are used in combination to score Principle 2. Scoring will be dependent on the health-related discipline and the information that is provided.

Best practice inclusions:

- Whether the account holder is adequately qualified to provide information will depend on the type of information provided. For example, it may be appropriate for someone without nutrition qualifications to provide general information about the importance of vegetable consumption. However, nutrition qualifications would be required for the provision of information about dietary intake for individuals with serious health conditions.
- As per Principle 1, if the account is held by a company or brand, the qualifications of the author of the health-related information should be clearly disclosed in the post or the social media profile.

| **Criteria** | **Score** |
| --- | --- |
| The account holder has clearly indicated their qualifications. The health-related information provided is within their scope of practice and they are adequately qualified to provide the information. | 4 |
| The account holder has indicated that they hold a health-related qualification. The information provided in the post may be outside of their scope of practice, however, it is general and low risk information. | 3 |
| The account holder has indicated that they hold a health-related qualification, however, the information in the post is outside of their scope of practice, they are not adequately qualified to provide the information and/or have provided insufficient detail about their qualifications to determine if they are adequately qualified. | 2 |
| The account holder has clearly indicated that they have no health-related qualifications and health-related information is being provided. | 1 |
| Insufficient or no information about the account holder’s qualifications are included on the social media profile and health-related information has been provided. | 0 |

**Principle 3: Action-orientated**

*Health-related information provided on social media should be action-orientated and include clear, succinct messages to support decision making and provide context for the consumer.*

**Scoring this principle**

The information used to score Principle 3 should come entirely from the social media post being evaluated.

Best practice inclusions:

- Health-related social media posts should be action-orientated and provide clear information about practical actions to improve health.
- Context should be provided for the consumer. For example, it should be clear from the information provided why a certain behaviour is important for health and who the information applies to.
- All important information about the health-related topic being discussed should be succinct and clear. Convoluted explanations and inclusion of irrelevant detail should be avoided.

| **Criteria** | **Score** |
| --- | --- |
| Clear, succinct information that provides comprehensive context for the consumer is provided. The information is likely to support consumer decision making. | 4 |
| Clear, succinct information is provided, however, some minor contextual details are missing. The information may support consumer decision making. | 3 |
| Somewhat clear and succinct information is provided, however, some contextual details are missing. It is unclear if the information will support consumer decision making. | 2 |
| The information provided lacks clarity and succinctness. A number of key contextual details are missing, and the information is not likely to support consumer decision making. | 1 |
| The information provided is lengthy, convoluted and provides no context for the consumer. The information is likely to cause confusion rather than support decision making. | 0 |

**Principle 4: Financial Disclosure**

*Sponsorship, advertising, funding arrangements, financial support or any potential conflicts of interest should be fully disclosed in a prominent and clear manner.*

**Scoring this principle:**

If a post has been sponsored, paid for, contains advertising and/or a product that has been gifted this needs to be clearly and prominently disclosed in the social media post. Ongoing financial support and conflicts of interest should be disclosed on the social media profile. For example, ongoing sponsorship from a brand or company or ongoing funding from a funding body should be disclosed within a social media profile. When scoring Principle 4, the social media profile and the social media post being evaluated should both be considered.

Due to the rapidly evolving social media marketing industry, the information outlined below may be subject to change. Before using this tool, it is recommended that current social media advertising guidelines relevant to the country of interest are referred to.

Best practice inclusions:

- Advertising on social media is often purposefully covert.(1) Look for the tagging of brands and presence of certain products in the absence of financial disclosures.
- The hashtag #ad or #sponsored are often included in posts by influencers to disclose that the post is advertising.(1) If products have been supplied for free #gifted can be used.(1)
- Advertising hashtags can appear prominently at the start of the post but are often hidden in the post comments or amongst many other hashtags at the end of the post.(1, 2)
- For social media platforms that use hashtags inclusion of #ad or #sponsored is the minimum required disclosure for advertising.(1)
- Hashtags such as #Collab, #Ambassador, #PaidPartner or hashtags including brand names can be used in addition to but not instead of #ad or #sponsored.(2)
- Some social media platforms include advertising disclosure tools within the platform. If available, these should be used for sponsored posts.(1)
- For video content financial disclosures should be made in the video and the caption or description.(1)
- Most brands have one or more social media page that they use to market their products. Such social media pages are not required to disclose that their posts contain advertising for their products because this is strongly implied, unlike discreet product placement on influencer accounts.(2)
- Some social media platforms allow brands to tag products. Product tagging provides information about the product and its price within the social media platform and links to the brand’s website where the product can be purchased. This is an example of prominent and clear advertising.
- A statement in the social media profile about sponsorship or advertising never being accepted is an example of a clear and prominent financial disclosure.

| **Criteria** | **Score** |
| --- | --- |
| Financial disclosures are clearly and prominently displayed on the social media profile and/or sponsored post and comply with relevant sponsorship and advertising guidelines. | 4 |
| Financial disclosures are clearly displayed on the social media profile and/or sponsored post, however, disclosures are not prominently positioned and comply with some but not all relevant sponsorship and advertising guidelines. | 3 |
| Financial disclosures are made on the social media profile and/or sponsored post, however, they lack clarity and are not prominently positioned. Few components of relevant sponsorship and advertising guidelines are met.  OR  No financial disclosures have been made in the profile or post, however, the information in the post does not mention a particular product/brand and is unlikely to be sponsored. | 2 |
| Financial disclosures are made on the social media profile and/or sponsored post, however, they are ambiguous, difficult to locate and do not comply with relevant advertising and sponsorship guidelines. | 1 |
| Social media profile does not contain any financial disclosures or information about potential conflicts of interest. Social media post has product or brand tagged and/or featured but does not include financial disclosures. | 0 |

**Principle 5: Attribution**

*Health-related information on social media should include clear references and hyperlinks to the original source(s) of information used to compile the post.*

**Scoring this principle**

Attribution information should be included in the social media post being evaluated, rather than included on the social media profile. Principle 5 assesses the presence of references to the original sources of information, rather than the quality and appropriateness of the sources, which is assessed under Principle 6.

Best practice inclusions:

- There may be rare instances that original health-related information can be shared without the need for citations. For example, recipes, workouts, or meditations that have been developed by the account holder that are not accompanied by claims about associated health benefits. In such instances it should be indicated that the content is original.
- It should be clear when the original source of information was published.
- If all references cannot fit into the social media post, a link to the references and further information should be provided.
- Certain social media platforms may be subject to word or character limits. In any case, the original source should be easily located from the information provided in the social media post.

| **Criteria** | **Score** |
| --- | --- |
| Clear references and hyperlinks to the original source(s) of information have been included. The year that the information was produced is also indicated. | 4 |
| Attribution information about the original source(s) of information have been included, however, hyperlinks and/or the year that the information was produced are missing. Sufficient detail is included to easily locate the original source(s) of information. | 3 |
| Attribution information about the original source(s) of information have been included, however, key details are missing. It is difficult to locate the original source(s) from the information provided. | 2 |
| An attempt at providing attribution information has been made. However, insufficient detail has been provided to locate the original source(s) of information. | 1 |
| Health-related information has been provided in a social media post and no information about the original source of the information has been included. | 0 |
| Post is compiled of original material that does not require citations. No claims are made. | N/A |

**Principle 6: Balance & Justifiability**

*Health-related information provided on social media that includes claims relating to the benefits/performance of a particular treatment, product, service or behaviour should be balanced, unbiased and supported by appropriate and quality evidence.*

**Scoring this principle:**

Information included within the social media post being evaluated should be used to score Principle 6. The presence or absence of citations is evaluated under Principle 5 and when scoring Principle 6, the appropriateness and quality of the evidence provided is assessed. When citations are provided, this will also require a brief evaluation of the quality of the source of evidence.

Best practice inclusions:

- If a claim is indicated to be based on research, a peer-reviewed journal article should be cited.
- High quality and appropriate evidence should be used. For example, systematic reviews, meta-analyses and government or peak body guidelines are appropriate to use when claims are made.
- Low quality and inappropriate evidence should be avoided. For example, use of animal studies to evidence effects in humans, use of inconclusive studies or studies with very small sample sizes.
- Limitations, areas of uncertainty and contrasting findings should be clearly discussed.
- The use of shock tactics and causative language should be avoided. For example, claims about effectiveness or performance should avoid terms such as ‘guarantee’, and ‘cure’.(3)
- If there are no claims about the performance or health outcomes associated with a treatment, product, service or health behaviour, Principle 6 should be marked as not applicable.

| **Criteria** | **Score** |
| --- | --- |
| Information about a treatment, product, service or behaviour is provided and is balanced, unbiased and supported by appropriate and quality evidence. | 4 |
| Information about a treatment, product, service or behaviour is provided and is mostly balanced, unbiased and supported by appropriate evidence. Some additional detail would improve the balance and justifiability of the information. | 3 |
| Information about a treatment, product, service or behaviour is provided and is somewhat balanced and supported by evidence. The quality and appropriateness of the evidence is unclear and additional detail is required to justify the claims made. | 2 |
| Information about a treatment, product, service or behaviour is provided and is mostly unbalanced and biased. The author may have made an attempt at providing justification, however, it is incomplete and is not supported by appropriate and quality evidence. | 1 |
| Information about a treatment, product, service or behaviour is provided in a biased fashion. One-sided claims and about benefits and performance are made and are not supported by appropriate or quality evidence. | 0 |
| Information about a treatment, product, service or behaviour is not provided. | N/A |

**Principle 7: Risks & Benefits**

*Health-related information provided on social media about a particular treatment, product, service or behaviour should clearly outline associated risks and benefits.*

**Scoring this principle**

The information required to score Principle 7 should be included entirely within the social media post. This principle will not always be relevant to evaluate.

Best practice inclusions:

- If the associated risks and/or benefits are not known, it should be clearly indicated.
- If there are no known risks, this should be clearly identified, rather than omitting information about risks.
- The information provided should describe how choices related to the treatment, product, service or health behaviour may impact overall quality of life.
- When information about a treatment, product, service or health behaviour is not provided Principle 7 should be marked as not applicable.

| **Criteria** | **Score** |
| --- | --- |
| Information about a treatment, product, service or behaviour is provided and the associated risks and benefits are clearly and comprehensively outlined. | 4 |
| Information about a treatment, product, service or behaviour is provided and the associated risks and benefits are outlined, however, some minor details are missing. | 3 |
| Information about a treatment, product, service or behaviour is provided and some information about associated risks and benefits are provided, however, key details are missing. | 2 |
| Information about a treatment, product, service or behaviour is provided but only information about the associated benefits or risks are outlined. Information about either risks or benefits is completely missing. | 1 |
| Information about a treatment, product, service or behaviour is provided but associated risks and benefits are not mentioned. | 0 |
| Information about a treatment, product, service or behaviour is not provided. | N/A |

**Principle 8: Privacy**

*Health-related information on social media should respect principles of privacy and confidentiality.*

**Scoring this principle**

Principle 8 should be scored based on the information provided in a social media post. This principle will not be relevant to assess for all social media posts.

Best practice inclusions:

- If information, images or videos of others are shared, they are shared with permission.
- Examples of identifiable information include images or footage that show the client or patient’s face or “tagging” a client or patient’s social media account in a post.
- Social media posts that do not include any pictures, information or reference to a patient or client are not relevant to assess and should be marked as not applicable.

| **Criteria** | **Score** |
| --- | --- |
| Health-related information about a client or patient is provided including a statement indicating that consent has been provided by the individual. No identifying information has been included. | 4 |
| Health-related information about a client or patient is provided including a statement indicating that consent has been provided by the individual. Some identifying information has been provided, however, the identity of the individual is difficult to determine. | 3 |
| Health-related information about a client or patient is provided including a statement indicating that consent has been provided by the individual. The individual is clearly identifiable. | 2 |
| Health-related information about a client or patient provided with no mention of permission. No identifying information has been included. | 1 |
| Health-related information about a client or patient provided with no mention of permission. The individual is clearly identifiable. | 0 |
| The social media post does not include any pictures, information or reference to a patient or client. | N/A |

**Principle 9: Complementary Information**

*Health-related information provided on social media should provide support for individuals’ relationships with their doctor and other professional healthcare providers and should not be designed to replace such relationships. Support for discussion of options with the individuals’ healthcare provider should be included in posts containing health-related information.*

**Scoring this principle**

Principle 9 should be scored based on the information contained within the social media post being evaluated. There may be some instances in which this principle is not relevant to evaluate.

Best practice inclusions:

- Social media posts that contain information about particular health conditions, treatments or health-behaviours that can have considerable impacts on health should clearly disclose that decisions should be discussed with relevant professional healthcare providers.
- When information about a health condition, treatment, product, service or health behaviour is not provided Principle 9 does not need to be evaluated and should be marked as not applicable.
- If a general practitioner is the most relevant professional to see about the issue or topic being discussed then it is appropriate to recommend as such.

| **Criteria** | **Score** |
| --- | --- |
| Information about a health condition, treatment, product, service or behaviour is provided and is complementary. Strong support for individuals’ relationships with professional healthcare provider(s) and encouragement for discussion of choices with professional healthcare provider(s) is included. The specific healthcare professional(s) who should be consulted are identified (e.g., dermatologist, physiotherapist). | 4 |
| Information about a health condition, treatment, product, service or behaviour is provided and is complementary. Moderate support for individuals’ relationships with professional healthcare providers is included. It is suggested that choices should be discussed with a healthcare provider, however, relevant professions are not identified. | 3 |
| Information about a health condition, treatment, product, service or behaviour is provided and is somewhat complementary. A generic disclaimer that choices should be discussed with a doctor/general practitioner is included. | 2 |
| Information about a health condition, treatment, product, service or behaviour is provided and is not complementary. A generic disclaimer that individuals with health conditions should discuss choices with a doctor/general practitioner is included. | 1 |
| Information about a health condition, treatment, product, service or behaviour is provided and is not complementary. It is implied that there is no need to discuss decisions relevant to the information provided with a professional healthcare provider and the information replaces the relationship between the consumer and their healthcare provider. | 0 |
| Information about a health condition, treatment, product, service or behaviour is not provided. | N/A |

**Principle 10: Referrals & Support**

*Health-related information provided on social media should include referrals to additional sources of support and information.*

**Scoring this principle:**

Principle 10 should be scored based on the information provided within the social media post of interest. Additional sources of support and information should be provided within the social media post. If referrals are provided, it is necessary to briefly assess their relevance to the information provided.

Best practice inclusions:

- Where possible, direct hyperlinks to resources and additional sources of support and information should be included.
- It is best practice for referrals to further information or support to be provided within the social media post. However, links and referrals can also be supplied within the social media profile or bio. In this instance, no more than a 2 should be awarded.
- Social media posts that mention mental health should always provide the contact details for a mental health support service within the post.

| **Criteria** | **Score** |
| --- | --- |
| Referrals to relevant and comprehensive sources of additional support and information are provided within the social media post. Direct hyperlinks to all resources are provided. | 4 |
| Referrals to relevant sources of additional support and information are provided within the social media post, however, referrals could be more relevant and/or comprehensive. Direct hyperlinks to resources may or may not be provided. | 3 |
| Referrals to additional support and information are provided, however, they lack relevance or comprehensiveness. Referrals may be contained within the social media post or profile and direct hyperlinks to resources may or may not be provided. | 2 |
| Minimal referral to additional sources of information and support are provided within the social media post or profile. Resources lack relevance and comprehensiveness and no direct hyperlinks are provided. | 1 |
| No referrals to additional information or supports have been provided. | 0 |

**Principle 11: Readability & Comprehensibility**

*Health-related information on social media should avoid the use of technical language and medical jargon. Plain language should be used and information should be easily understood by the general public.*

**Scoring this principle**

The information provided within a social media post should be used to score Principle 11. If a social media post contains a video and accompanying text, both components should be independently assessed, and the average score should be included as the overall score for this principle. Written information and spoken information in a video should each be scored according to the following criteria.

**Written text**

Readability should be objectively assessed using the Flesch Reading Ease formula, which is inbuilt to Microsoft Word. The Flesch Reading Ease formula will calculate a score for Flesch-Kincaid Grade Level, which corresponds to the grade reading level. For example, a Flesch-Kincaid Grade Level score of seven indicates that the text is written at a Grade Seven reading level. Instructions on how to produce the Flesch-Kincaid Grade level score are outlined below.

Best practice inclusions:

- Written information should be written at a Grade Five reading level.
- It is common for text to be contained within an image. For example, screen shots of Twitter posts are often shared on Instagram. When this occurs, the text included in the image and the text included in the caption should be analysed using the instructions outlined below.


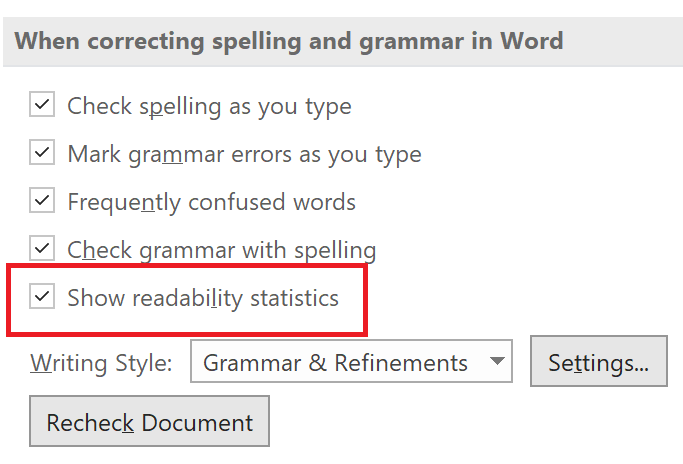


To allow Microsoft Word to assess readability the settings must be correct. To arrange the settings:

1. In Microsoft Word go to ***File 🡪 Options 🡪 Proofing***.
2. Under the ***Spelling and Grammar*** heading select the ***Show readability statistics*** box, pictured in Figure 1.
3. Click ***Okay*** to save settings.


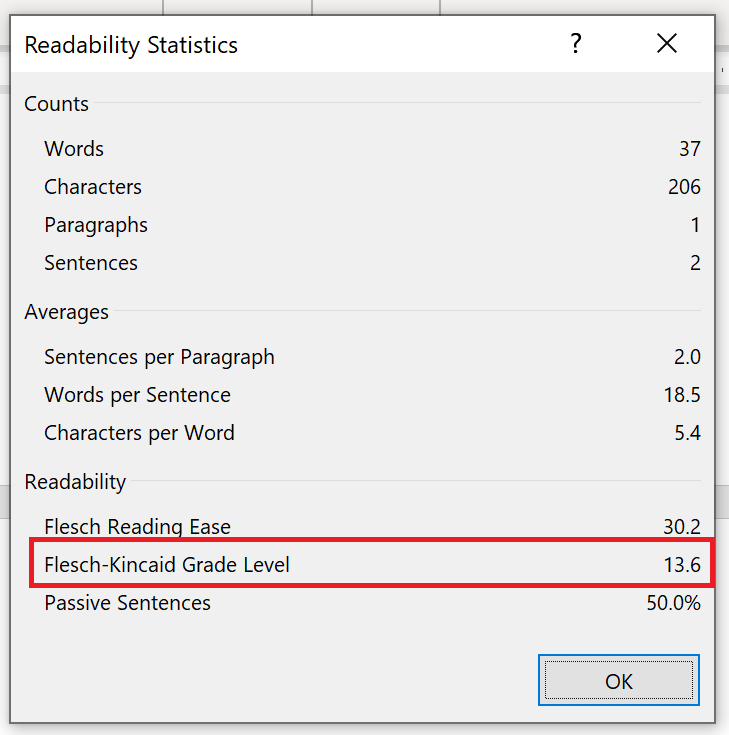
Figure 1: readability statistics settings

To generate a Flesch-Kincaid Grade Level:

1. Highlight the text to be analysed.
2. Go to the ***Review*** tab and select ***Spelling & Grammar***.
3. If any spelling or grammar issues are picked up select ***Ignore once*** for each issue.
4. A text box will appear that will contain the Flesch-Kincaid Grade Level, pictured in Figure 2.

Figure 2: Flesch-Kincaid Grade Level

| **Criteria** | **Score** |
| --- | --- |
| Flesch-Kincaid Grade Level of ≤ 5 | 4 |
| Flesch-Kincaid Grade Level of 5.1 – 8 | 3 |
| Flesch-Kincaid Grade Level of 8.1 – 10 | 2 |
| Flesch-Kincaid Grade Level of 10.1 – 11.9 | 1 |
| Flesch-Kincaid Grade Level of ≥ 12 | 0 |

**Video content**

Although content in videos does not require reading skills, the information should be easily understood by the audience and technical language should be avoided.

Best practice inclusions:

- Verbal communication in video content should use plain language i.e. the information should be easily understood the first time it is heard.(4)
- In some instances, technical terms may be appropriate to include, however, their meaning should be explained in plain language. If technical terms are not required they should be avoided altogether.

| **Criteria** | **Score** |
| --- | --- |
| Plain language is used for the entirety of the video. Technical terms are only included if they are essential and, if included, they are explained in simple terms. The information would be easily understood by non-specialist audiences. | 4 |
| Plain language is used for the majority of the video. A small number of technical terms are included unnecessarily, however, they are explained in simple terms. The information is likely to be understood by non-specialist audiences. | 3 |
| Plain language is used in approximately half of the video. Technical terms are included unnecessarily and are explained in somewhat simple terms. It is likely that non-specialist audiences would find some of the information difficult to understand. | 2 |
| Most of the language used in the video is technical. Some but not all technical terms are explained. It is likely that non-specialist audiences would find most of the information difficult to understand. | 1 |
| Technical language and jargon are used throughout the entire video. The information would be difficult for non-specialist audiences to understand. | 0 |

**Principle 12: Accessibility**

*Health-related information provided on social media should be accessible to vision- and hearing-impaired individuals.*

**Scoring this principle**

Principle 12 should be assessed based on the content contained within the social media post, rather than the social media profile. Where relevant, social media posts that include images should provide alternative text* in the caption and videos should include closed captions. Alternative text may not be automatically displayed, and a screen reader app or web browser add-in designed for vision-impaired individuals may need to be installed to evaluate alternative text. This will depend on the social media platform being used.

*Alternative text, also known as ‘alt text’ provides a description of the appearance and/or function of an image.

Best practice inclusions:

- Alternative text is often automatically generated by a social media platform’s artificial intelligence, which attempts to identify objects within an image. Automatically generated alternative text can often contain inaccuracies.(5)
- Closed captions for video can also be automatically generated and often contain inaccuracies.(6)
- Camel case, where the first letter of each word is capitalised, should be used in hashtags that contain multiple words, for example, #HealthyLifestyle. Capitalising the first letter of each word within the hashtag makes it easier for people with low vision to read and are more consumable by screen readers.(7)
- Emojis and emoticons should be used sparingly and with spaces in between them to improve the experience of those using a screen reader.(7)
- Formatting of text within social media posts should be avoided because formatted words may not be picked up by screen readers or the formatting may be read aloud by screen readers. For example, ‘**bold**’ may be read aloud as ‘bold bold’.(8)
- Alternative text is sometimes contained at the end of a post’s written caption with a heading that says, ‘image description’, ‘alternative text’ or ‘alt text’. If this occurs, the description included should also be assessed.
- If an image contains text, the image-based text should be included in the alternative text or caption.

| **Criteria** | **Score** |
| --- | --- |
| The social media post is highly accessible to vision- and/or hearing-impaired individuals. Accessibility considerations are appropriate, comprehensive, highly accurate and descriptive. | 4 |
| Relevant accessibility considerations are included and are mostly accurate, comprehensive and detailed. Some minor inaccuracies are present and/or minor details omitted. Vision- and hearing-impaired individuals would be able to access the majority of information in the post. | 3 |
| Some relevant accessibility considerations are included, however, they contain inaccuracies, are incomplete and/or lack detail. Vision- and/or hearing-impaired individuals would have difficulty accessing some of the information in the post. | 2 |
| Few relevant accessibility considerations are included, however, they are inaccurate, incomplete, lack detail and/or are missing. Vision- and/or hearing-impaired individuals would have difficulty accessing most of the information in the post. | 1 |
| The social media post is not accessible to vision- and/or hearing-impaired individuals. The social media post contains video, image, hashtags and/or emojis and no accessibility considerations have been included. | 0 |
| The social media post does not include video, images, emojis or hashtags. | N/A |

**Principle 13: Images**

*Images included in health-related social media posts should be visually appealing and reflect rather than contradict the information provided in the post.*

**Scoring this principle**

When relevant Principle 13 should be assessed on the image contained in a social media post and the accompanying information contained in the text component of the post. This principle only needs to be evaluated when posts include images.

Best practice inclusions:

- Click-bait images that are included to attract attention but have no relevance to the other information contained in the social media post should be avoided.
- Social media posts that do not contain images are not relevant to assess and should be marked as not applicable.
- Posts that contain images that only contain text, for example, a screenshot of a tweet that does not contain images, should also be marked as not applicable.

| **Criteria** | **Score** |
| --- | --- |
| Images are visually appealing and appropriately reflect the information contained in the post. | 4 |
| Images are fairly visually appealing and appropriately reflect the information contained in the post. | 3 |
| Images are not visually appealing but appropriately reflect the information contained in the post or are visually appealing but are not related to the information contained in the post. | 2 |
| Images are not visually appealing and are not related to the information contained in the post. | 1 |
| The image(s) directly contradict the health-related information included in the social media post. | 0 |
| The social media post does not include images. | N/A |

**References:**

1. Australian Influencer Marketing Council. Australian Influencer Marketing Code of Practice. Sydney; 2020.

2. Australian Association of National Advertisers. Clearly Distinguishable Advertising. Sydney; 2018.

3. Robillard JM, Jun JH, Lai J-A, Feng TL. The QUEST for quality online health information: validation of a short quantitative tool. BMC Medical Informatics and Decision Making. 2018;18(1):87.

4. Wicklund K, Ramos K. Plain Language: Effective Communication in the Health Care Setting. Journal of Hospital Librarianship. 2009;9(2):177-85.

5. Sharma R. Why you should add Instagram Alt Text to your posts: Tailwind Blog; 2021 [Available from: <https://www.tailwindapp.com/blog/instagram-alt-text>.

6. Leetaru K. Facebook's terrible autimatic video captions are no laughing matter: Forbes; 2019 [Available from: <https://www.forbes.com/sites/kalevleetaru/2019/04/19/facebooks-terrible-automatic-video-captions-are-no-laughing-matter/?sh=11be3a87798f>.

7. Princeton University. Social Media Accessibility Guidelines: Princeton University; 2021 [Available from: <https://accessibility.princeton.edu/guidelines/social-media#platforms>.

8. Roselli A. Improving your tweet accesibility 2018 [Available from: <https://adrianroselli.com/2018/01/improving-your-tweet-accessibility.html#Captions>.
